# Measuring mortality due to HIV-associated tuberculosis among adults in South Africa: Comparing verbal autopsy, minimally-invasive autopsy, and research data

**DOI:** 10.1371/journal.pone.0174097

**Published:** 2017-03-23

**Authors:** Aaron S. Karat, Mpho Tlali, Katherine L. Fielding, Salome Charalambous, Violet N. Chihota, Gavin J. Churchyard, Yasmeen Hanifa, Suzanne Johnson, Kerrigan McCarthy, Neil A. Martinson, Tanvier Omar, Kathleen Kahn, Daniel Chandramohan, Alison D. Grant

**Affiliations:** 1 Department of Clinical Research, London School of Hygiene & Tropical Medicine, London, United Kingdom; 2 The Aurum Institute, Johannesburg, South Africa; 3 Department of Infectious Disease Epidemiology, London School of Hygiene & Tropical Medicine, London, United Kingdom; 4 School of Public Health, Faculty of Health Sciences, University of the Witwatersrand, Johannesburg, South Africa; 5 Foundation for Professional Development, Pretoria, South Africa; 6 Division of Public Health, Surveillance and Response, National Institute for Communicable Disease of the National Health Laboratory Service, Johannesburg, South Africa; 7 Perinatal HIV Research Unit, and Medical Research Council Soweto Matlosana Collaborating Centre for HIV/AIDS and TB, University of the Witwatersrand, Johannesburg, South Africa; 8 Johns Hopkins University Center for TB Research, Baltimore, United States of America; 9 Department of Science and Technology / National Research Foundation Centre of Excellence for Biomedical TB Research, University of the Witwatersrand, Johannesburg, South Africa; 10 Department of Anatomical Pathology, National Health Laboratory Service and University of the Witwatersrand, Johannesburg, South Africa; 11 MRC/Wits Rural Public Health and Health Transitions Research Unit (Agincourt), School of Public Health, Faculty of Health Sciences, University of the Witwatersrand, Johannesburg, South Africa; 12 INDEPTH Network, Accra, Ghana; 13 Epidemiology and Global Health Unit, Department of Public Health and Clinical Medicine, Umeå University, Umeå, Sweden; 14 Department of Disease Control, London School of Hygiene & Tropical Medicine, London, United Kingdom; 15 Africa Health Research Institute, School of Nursing and Public Health, University of KwaZulu-Natal, Durban, South Africa; Médecins Sans Frontières (MSF), SOUTH AFRICA

## Abstract

**Background:**

The World Health Organization (WHO) aims to reduce tuberculosis (TB) deaths by 95% by 2035; tracking progress requires accurate measurement of TB mortality. International Classification of Diseases (ICD) codes do not differentiate between HIV-associated TB and HIV more generally. Verbal autopsy (VA) is used to estimate cause of death (CoD) patterns but has mostly been validated against a suboptimal gold standard for HIV and TB. This study, conducted among HIV-positive adults, aimed to estimate the accuracy of VA in ascertaining TB and HIV CoD when compared to a reference standard derived from a variety of clinical sources including, in some, minimally-invasive autopsy (MIA).

**Methods and findings:**

Decedents were enrolled into a trial of empirical TB treatment or a cohort exploring diagnostic algorithms for TB in South Africa. The WHO 2012 instrument was used; VA CoD were assigned using physician-certified VA (PCVA), InterVA-4, and SmartVA-Analyze. Reference CoD were assigned using MIA, research, and health facility data, as available. 259 VAs were completed: 147 (57%) decedents were female; median age was 39 (interquartile range [IQR] 33–47) years and CD4 count 51 (IQR 22–102) cells/μL. Compared to reference CoD that included MIA (n = 34), VA underestimated mortality due to HIV/AIDS (94% reference, 74% PCVA, 47% InterVA-4, and 41% SmartVA-Analyze; chance-corrected concordance [CCC] 0.71, 0.42, and 0.31, respectively) and HIV-associated TB (41% reference, 32% PCVA; CCC 0.23). For individual decedents, all VA methods agreed poorly with reference CoD that did not include MIA (n = 259; overall CCC 0.14, 0.06, and 0.15 for PCVA, InterVA-4, and SmartVA-Analyze); agreement was better at population level (cause-specific mortality fraction accuracy 0.78, 0.61, and 0.57, for the three methods, respectively).

**Conclusions:**

Current VA methods underestimate mortality due to HIV-associated TB. ICD and VA methods need modifications that allow for more specific evaluation of HIV-related deaths and direct estimation of mortality due to HIV-associated TB.

## Introduction

Data on causes of death (CoD) are essential in guiding public health strategy and research agendas. In South Africa in 2014, HIV prevalence was 18.9% among individuals aged 15–49 years [1] and there were an estimated 72,000 deaths due to HIV-associated tuberculosis (TB) [2]. However, methods to measure CoD, particularly HIV-associated TB, do not provide sufficiently accurate estimates [3]. Autopsy studies have typically investigated small numbers of deaths and mostly included individuals dying in hospitals [4–6]; death certification has consistently been shown to correspond poorly with retrospective clinical and autopsy diagnoses [7–9]; and verbal autopsy (VA), used to estimate CoD at a population level in many areas with poor civil registration systems, differentiates poorly between deaths due to HIV-associated TB and other HIV-associated causes [10–12]. These difficulties are compounded by the way deaths due to HIV-associated TB are coded by the International Classification of Diseases (ICD), which counts them as ‘HIV-related’ [13]. The World Health Organization (WHO) presents TB mortality data separately for HIV-negative and HIV-positive individuals. However, due to the coding issues described above, all estimates of mortality due to HIV-associated TB are generated indirectly through mathematical modelling that uses country-level data on TB incidence and case-fatality ratios [14,15]; WHO itself states the ‘urgent need’ for direct measurement of HIV-associated TB mortality [14]. An aim of the WHO ‘End TB’ strategy is to reduce TB deaths by 95% by 2035 [16].

Verbal autopsy is a structured interview with the family or carer of a deceased individual, carried out by a lay-interviewer. A standardised VA instrument has been available from WHO since 2007 [17] and is now used globally at health and socio-demographic surveillance system (HDSS) sites. In areas with poor civil registration systems, VA data are used to generate estimates of national and regional mortality [18] which may then be used to influence health policy. The last 10 years have seen the development of several automated methods (computer-coded VA [CCVA]) which, it is hoped, will eventually replace the expensive and time-consuming physician-certified VA (PCVA) [19,20]. Numerous studies have attempted to validate VA, but the vast majority compare VA CoD to clinical CoD derived from physician review of hospital or clinic records, a gold standard of variable quality and consistency [21–24]. There have been few attempts to compare VA CoD to CoD derived from research-quality data or from pathological autopsy, which remains the highest standard for assigning CoD.

More accurate estimates of HIV-associated mortality are particularly needed in areas of high HIV prevalence, where civil registration systems are often weak [14]. This study, nested within two large studies of HIV-positive adults in South Africa (‘TB Fast Track’ [25] and ‘XPHACTOR’), aimed to compare VA CoD to reference-standard CoD derived from clinical, research, and minimally-invasive autopsy (MIA) data.

## Methods

### Setting

HIV-positive adults (aged ≥18 years) were recruited to one of two studies of TB/HIV conducted in South Africa. The first, ‘TB Fast Track’, was a pragmatic trial of empirical TB treatment in ambulant HIV-positive adults enrolled in primary care with CD4 count ≤150 cells/μL. Participants were eligible if not on antiretroviral therapy (ART) or TB treatment at the point of enrolment and were followed up for a minimum of six months [25]; MIA and VAs were conducted for as many decedents as possible. The second, ‘XPHACTOR’, was an interventional cohort study investigating the use of Xpert^®^ MTB/RIF in a systematic sample of HIV-positive adults attending out-patient clinics for HIV care; participants were followed up for at least three months and VAs were conducted for as many decedents as possible, beginning about half-way through the study’s follow-up.

### Data collection

#### Verbal autopsy

All VA interviews were conducted by trained lay researchers at the home of the family/carers, or at another location of their choosing, one to twelve months after the death of the study participant, as recommended by WHO. Written informed consent for VA was obtained from respondents; all interviews were conducted using the WHO 2012 VA instrument, with additional questions around treatment for TB and HIV, health beliefs, and health service use added by the study team.

#### Clinical

Data used to inform reference-standard CoD were separated into three categories: those collected from routine clinic and/or hospital records by a trained lay researcher, research nurse, or physician using standardised paper forms, labelled *operational*; those collected by members of a clinical research team due to involvement in a parent study, including results of investigations retrieved from the national health laboratory service (NHLS) database, labelled *research*; and those collected through MIA, carried out on TB Fast Track decedents as soon as possible after death, labelled *autopsy* (S1 Fig). Detailed MIA methods and a description of the consent process have previously been described [26]. The procedure involved core biopsies of liver, spleen, and lungs; aspiration of cerebrospinal fluid (CSF), blood, and urine; sampling of naso- and oro-pharyngeal secretions; and broncho-alveolar lavage (BAL) by insertion of a nasogastric tube into the trachea directed toward the lungs through a cricothyroid incision. Laboratory testing of post-mortem specimens included liquid culture for mycobacteria; Xpert^®^ MTB/RIF; microscopy and aerobic culture; molecular testing for a range of bacteria and viruses; and histological examination.

### Data interpretation

#### Verbal autopsy

VA data included in the WHO 2012 VA instrument (i.e., excluding data from study-specific added questions around ART use, treatment for TB, health beliefs, or health service use) were interpreted using both physician-certified verbal autopsy (PCVA) and computer-coded verbal autopsy (CCVA) methods. Using a PCVA method based on WHO recommendations, similar to that used at the Medical Research Council/Wits-Agincourt HDSS site, South Africa [21], two physicians, blinded to all other clinical information, independently reviewed all available VA data, including the free narratives, and separately assigned CoD using ICD-10 and study-defined codes (detailed below). Assigned CoD were compared and, where there were discrepancies in either immediate, underlying, or study-defined CoD, the cases were discussed by the two physicians, aiming for consensus. If a consensus could not be reached, the data were provided to a third physician who reviewed them independently. If the CoD assigned by physician 3 matched that assigned by physicians 1 or 2, it was considered the final CoD; if no consensus was reached after review by three physicians, the individual was assigned an ‘indeterminate’ CoD.

VA data were also processed by two CCVA methods. The first, InterVA-4 (www.interva.net), uses Bayesian probabilities to assign each decedent up to three CoD, each with an associated ‘likelihood’ expressed as a percentage probability; cause-specific mortality fractions (CSMFs) can be generated that combine all individual causes and likelihoods [27,28]. The model allows for user modification of two baseline variables: prevalence of malaria and HIV, each of which can be set to ‘very low’, ‘low’, or ‘high’. The second method, SmartVA-Analyze (http://www.healthdata.org/verbal-autopsy/tools), uses the Tariff 2.0 system to assign each decedent one of 34 CoD [29,30]. SmartVA-Analyze also allows for the inclusion of data from the narrative section of the VA instrument and from healthcare records examined during the interview. Data were mapped from the WHO 2012 instrument to the 2014 framework for InterVA-4 and to the Population Health Metrics Research Consortium (PHMRC) full instrument for SmartVA-Analyze; free narrative and healthcare data were not provided to either software. Both CCVA methods assign CoD to lists of grouped ICD-10 codes [31,32]; these were further grouped into seven major categories for analysis (S1 Table).

#### Clinical

In order to meaningfully compare results with other VA validation studies, at least two sets of reference CoD were assigned to each participant. Operational data were used to generate level one (L1) CoD, comparable to the gold standard used in the majority of VA validation studies (S1 Fig). Both operational and research data were used to generate level two (L2) CoD for all decedents, representing a higher gold standard than would normally be available for comparison to VA. Finally, operational, research, and autopsy data were used to generate level three (L3) CoD for decedents for whom these data were available. All parties involved in the assignment of reference CoD were blinded to VA data, parent study arm, and any narratives around death obtained from family members as part of the research process, which were considered too similar to VA. L1 and L2 CoD were assigned using the same method as PCVA but involved different physicians; cases were processed in batches of 40–50. For each batch, all decedents were required to have finalised L1 CoD before the physicians were exposed to any higher level data.

L3 CoD were assigned in a different manner. Once all decedents with autopsy data had been assigned L1 and L2 CoD, a panel was convened to assign L3 CoD. The panel was made up of two infectious disease physicians, a microbiologist, and a pathologist, all of whom had extensive knowledge of local epidemiology; it was blinded to VA data, TB Fast Track study arm, and narratives from family members. The panel reviewed all available clinical data and attempted to reach consensus on CoD. In cases where full consensus could not be reached, consensus among three panel members was considered sufficient; if opinion was evenly split, the decedent was assigned an ‘indeterminate’ CoD.

### Study-defined CoD

To differentiate HIV-associated TB from other HIV-associated causes, six broad study-defined CoD categories were constructed: TB in an HIV-positive individual; an HIV/AIDS-related cause, excluding TB; a cause unrelated to HIV in an HIV-positive individual; TB in an HIV-negative individual; a cause other than TB in an HIV-negative individual; and an indeterminate cause. As part of both PCVA and reference CoD processes, reviewers assigned each decedent ICD-10 and study-defined CoD, along with a probability of ‘definite’, ‘probable’, or ‘possible’. For reference CoD only, probabilities were based on pre-defined criteria (S2 Table). CCVA outputs were not reclassified into study-defined categories as they do not allow HIV-associated TB to be distinguished from other HIV/AIDS-related CoD.

### Data management and statistical analyses

VA quantitative data were entered directly into an online database (Mobenzi Technologies, Durban, South Africa) through a cell phone interface; narrative data were captured on paper. Data collected for reference CoD assignment were entered into EpiData v3.1 (The EpiData Association, Odense, Denmark) and data from the parent studies into a SQL database (Bytes Technology Group, Johannesburg, South Africa). InterVA-4.03 was used, with malaria prevalence set to ‘Low’ and HIV/AIDS to ‘High’; InterVA-4 CSMFs were generated by dividing the sum of the likelihoods of each cause category by the sum of likelihoods for all causes [27]. SmartVA-Analyze v1.1.1 was used, with ‘Malaria region’, ‘Health Care Experience’, and ‘Free text’ options deselected; CSMFs, including deaths with ‘Undetermined’ cause, were calculated after outputs were grouped further (S1 Table). The Mortality Medical Data System (MMDS) 2011 software package [33] was used to generate a single ‘underlying’ CoD from ICD-10 codes assigned by PCVA and clinical panels; CSMFs were calculated using ACME/TRANSAX output. All analyses were conducted using Stata v14 (StataCorp, College Station, TX, USA).

Two forms of agreement were measured: between CoD assigned to individual decedents; and between the proportion of deaths assigned to each cause category across the study population. Cohen’s kappa (Κ) was used to measure agreement between individual decedents and overall chance-corrected concordance (CCC) used for agreement between cause categories; 1 equated to perfect agreement and 0 to agreement no greater than chance. Lin’s concordance correlation coefficient (*ρ*_C_) and CSMF accuracy were calculated for population-level comparisons [34]. In line with previous uses of *ρ*_C_, a value of less than 0.90 was considered ‘poor’ agreement [35].

### Ethical considerations

Separate approvals were obtained for the parent studies and the sub-study from the human research ethics committees of the London School of Hygiene & Tropical Medicine and the University of the Witwatersrand. Beginning in August 2013, participants in TB Fast Track were asked to give written informed consent for MIA in the event of their death while undergoing follow-up as part of the parent study. If a participant who had given written consent died during follow-up, verbal agreement from the next of kin was obtained to proceed with MIA. For participants who were enrolled to TB Fast Track prior to August 2013 and died during follow-up, formal written consent to undertake MIA was sought from the next of kin. All VA respondents gave written informed consent for interview.

## Results

### Demographics

A total 3022 individuals were recruited to the TB Fast Track study between December 2012 and December 2014; 364 died after enrolment. XPHACTOR enrolled a total 3722 individuals between September 2012 and March 2014; 125 died after enrolment. Attempts were made to contact the families of all deceased individuals between August 2013 and October 2015: contact could not be made in 218/489 (44.6%) cases and respondents declined to participate in 12 (2.4%) cases; 212/364 (58.2%) TB Fast Track decedents and 47/125 (37.6%) XPHACTOR decedents had a VA completed; a total 259 VAs were conducted (S1 Fig).

Among the 259 decedents (Table 1), 147 (57%) were female; the median age was 39 (interquartile range [IQR] 33–47) years; the median CD4 count at enrolment into the parent study was 51 (IQR 22–102) cells/μL; 258 (99.6%) were black African; 248 (96%) were South African; and 203 (78%) were enrolled in a peri-urban area, as opposed to semi-rural. The median time from enrolment to death was 84 (IQR 39–184) days and from death to VA was 146 (IQR 82–290) days.

**Table 1:** Demographics for all decedents for whom a VA was conducted, stratified by parent study (n = 259).

Characteristic	All, n (%) or median (IQR)	TB Fast Track, n (%) or median (IQR)	XPHACTOR, n (%) or median (IQR)
N	259	212	47
Female	147 (57)	115 (54)	32 (68)
Age at death	39 (33–47)	39 (33–46)	43 (37–51)
CD4 count at enrolment* (cells/μL)	51 (22–102) (n = 257)	44 (19–88)	161 (42–335) (n = 45)
Black African	258 (99.6)	211 (99.5)	47 (100)
South African	248 (96)	203 (96)	45 (96)
Enrolled in a peri-urban area	203 (78)	156 (74)	47 (100)
Completed grade 12	85 (33)	69 (33)	16 (34)
Household income of ≤R2000 per month†	98 (49) (n = 202)	78 (49) (n = 159)	20 (47) (n = 43)
On ART at enrolment*	32 (12)	0	32 (68)
Previous treatment for TB	46 (18)	32 (15)	14 (30)
Time from enrolment* to death (days)	84 (39–184)	76 (33–168)	109 (70–344)
Time from death to VA (days)	146 (82–290)	142 (78–288)	170 (95–316)
Parent as VA respondent	75 (29)	61 (28)	14 (30)

* Refers to enrolment in the parent study (TB Fast Track or XPHACTOR)

^†^ 202/259 (80%) individuals were able to estimate household income

ART: antiretroviral therapy; IQR: interquartile range; R: South African Rand; TB: tuberculosis; VA: verbal autopsy

### Data availability and consistency in physician assignment

Of the 212 TB Fast Track decedents, 196 (92%) had clinic files available; 122 (58%) had hospital files available; all had research data available; 207 (98%) had data from the NHLS database available; and 34 (26%) had MIA conducted, a median five (IQR 3–6) days after death. Of the 47 XPHACTOR decedents, all had clinic files available; none had hospital files available; all had research data available; and 33 (70%) had data from the NHLS database available (S1 Fig).

In assigning L1 CoD, physicians were in agreement on all three of immediate, underlying, and study-defined causes for 129/259 (49.8%) decedents; in assigning L2, they agreed 138/259 (53.3%) times. Physicians assigning VA CoD agreed on all three causes for 90/259 (34.7%) decedents. If agreement was measured based only on study-defined causes, it improved to 146/259 (56.4%), 162 (62.5%), and 141 (54.4%) times for L1, L2, and PCVA CoD, respectively. All disagreements were resolved through discussion; arbitrating physicians were not required.

### Performance of VA against L3 reference standard

**Fig 1:**
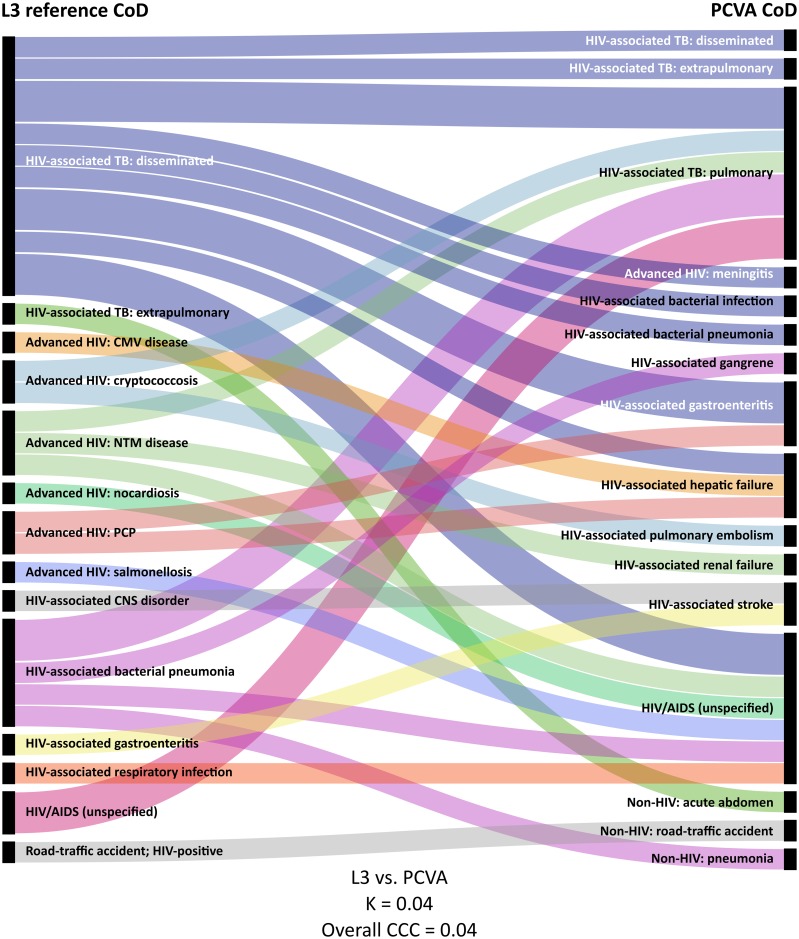
Alluvial* diagram showing CoD† as assigned by the clinicopathological panel (left) and PCVA (right); n = 34 [36]. *Each horizontal band represents one decedent. †Combined ‘immediate’ and ‘underlying’ ICD-10 CoD. ‡Comparison based on study-defined codes. AIDS: acquired immune deficiency syndrome; CCC: chance-corrected concordance; CMV: cytomegalovirus; CNS: central nervous system; CoD: cause of death; HIV: human immunodeficiency virus; K: Cohen’s kappa; L3: level three (using operational, research, and autopsy data); NCD: non-communicable diseases; NTM: non-tuberculous mycobacteria; PCP: *Pneumocystis* pneumonia; PCVA: physician-certified verbal autopsy; TB: tuberculosis.

**Fig 2:**
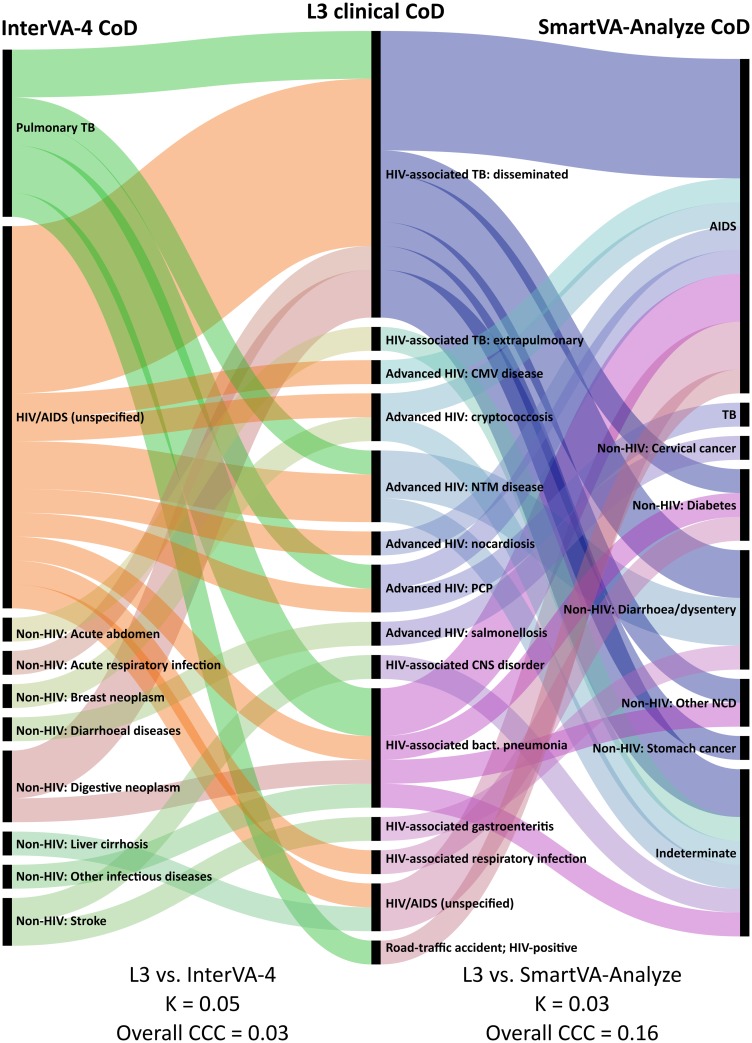
Alluvial* diagram showing CoD as assigned by clinicopathological panel† (centre), InterVA-4‡ (left) and SmartVA-Analyze (right); n = 34 [36]. *Each horizontal band represents one decedent. †Combined ‘immediate’ and ‘underlying’ ICD-10 CoD. ‡InterVA-4 CoD with highest associated likelihood. AIDS: acquired immune deficiency syndrome; CCC: chance-corrected concordance; CMV: cytomegalovirus; CNS: central nervous system; CoD: cause of death; HIV: human immunodeficiency virus; K: Cohen’s kappa; L3: level three (using operational, research, and autopsy data); NCD: non-communicable diseases; NTM: non-tuberculous mycobacteria; PCP: *Pneumocystis* pneumonia; PCVA: physician-certified verbal autopsy; TB: tuberculosis.

Thirty-four decedents underwent MIA, detailed results of which have previously been reported [26]: 18 (53%) were female; median age at death was 39 (IQR 33–44) years, and median CD4 count at enrolment to TB Fast Track was 34 (IQR 17–66) cells/μL. Using ICD-10 codes, 32/34 (94.1%) individuals were assigned an ‘HIV/AIDS-related’ reference CoD by the clinicopathological panel, compared to 25 (73.5%) assigned by PCVA, 47.1% assigned by InterVA-4, and 14 (41.2%) assigned by SmartVA-Analyze (Table 2). A ‘pulmonary TB’ CoD was assigned to 20.6% by InterVA-4 and to one (2.9%) decedent by SmartVA-Analyze; this was not assigned to any decedents by either PCVA or the clinical panel. All VA methods performed poorly when CoD assigned to individual decedents were compared to L3 (Figs 1 and 2): PCVA (K 0.13; CCC 0.22) performed better than the CCVA methods (K 0.05 and 0.03; overall CCC 0.01 and 0.16; for InterVA-4 and SmartVA-Analyze, respectively; Table 3). When compared at a population level, PCVA performed better (*ρ*_C_ 0.95; CSMF accuracy 0.79), but both CCVA methods were still sub-optimal (*ρ*_C_ 0.67 and 0.58; CSMF accuracy 0.49 and 0.43; for InterVA-4 and SmartVA-Analyze, respectively).

**Table 2:** Numbers and resultant CSMFs generated for grouped ICD-10 and study-defined CoD categories in those with autopsy data available, as assigned by the clinicopathological panel, PCVA, InterVA-4, and SmartVA-Analyze (n = 34).

CoD category	L3 reference standard, n (%)	VA method		
		PCVA, n (%)	InterVA-4, % (CSMF)*	SmartVA-Analyze, n (%)
**Grouped ICD-10**				
HIV/AIDS	32 (94.1)	25 (73.5)	47.1	14 (41.2)
PTB	0	0	20.6	1 (2.9)
Other infectious	1 (2.9)	5 (14.7)	8.8	5 (14.7)
Malignancy	0	0	11.8	2 (5.9)
Other NCD	0	3 (8.8)	11.8	5 (14.7)
External/traumatic or pregnancy-related	1 (2.9)	1 (2.9)	0	0
Indeterminate	0	0	0	7 (20.6)
**Study-defined**				
TB in an HIV-positive individual	14 (41.2)	11 (32.4)	-	-
HIV-related cause other than TB	15 (44.1)	17 (50.0)	-	-
Other cause in an HIV-positive individual	1 (2.9)	3 (8.8)	-	-
TB in an HIV-negative individual	0	0	-	-
Other cause in an HIV-negative individual	0	3 (8.8)	-	-
Indeterminate cause	4 (11.8)	0	-	-

* InterVA-4 CSMFs generated from all assigned CoD and associated likelihoods

AIDS: acquired immune deficiency syndrome; CoD: cause of death; CSMF: cause-specific mortality fraction; HIV: human immunodeficiency virus; ICD: International Classification of Diseases; L3: level three (operational, research, and autopsy data); NCD: non-communicable disease; PCVA: physician-certified verbal autopsy; PTB: pulmonary tuberculosis; TB: tuberculosis

**Table 3:** Summary of measures of performance of PCVA, InterVA-4, and SmartVA-Analyze in assigning CoD in ICD-10 or study-defined categories, compared to L3 (n = 34) and L2 (n = 259) reference standards.

CoD category and VA method	Compared to L3 reference standard (n = 34)				Compared to L2 reference standard (n = 259)			
	Individual measures‡		Population measures		Individual measures‡		Population measures	
	Cohen’s kappa (95% CI)	Overall CCC	*ρ*_C_ (95% CI)	CSMF accuracy	Cohen’s kappa (95% CI)	Overall CCC	*ρ*_C_ (95% CI)	CSMF accuracy
Grouped ICD-10*								
PCVA	0.13 (0–0.47)	0.22	0.95 (0.86–0.98)	0.79	0.06 (0–0.13)	0.14	0.93 (0.69–0.99)	0.78
InterVA-4	0.05 (0–0.08)	0.01	0.67 (0.38–0.84)	0.49	0.08 (0.03–0.11)	0.06	0.78 (0.44–0.93)	0.61
SmartVA-Analyze	0.03 (0–0.11)	0.16	0.58 (0.26–0.78)	0.43	0.05 (0.04–0.08)	0.15	0.52 (0.06–0.79)	0.57
**Study-defined**†								
PCVA	0.04 (0–0.20)	0.04	0.91 (0.51–0.99)	0.79	0.06 (0.04–0.08)	0.08	0.70 (0–0.95)	0.71

* Overall CCC for grouped ICD-10 calculated across seven possible CoD categories

^†^ Overall CCC for study-defined calculated across six possible CoD categories

^‡^ For InterVA-4, measures of individual agreement calculated using assigned CoD with highest associated likelihood

CCC: chance-corrected concordance; CI: confidence interval; CoD: cause of death; CSMF: cause-specific mortality fraction; ICD: International Classification of Diseases; L2: level two (operational and research data); L3: level three (operational, research, and autopsy data); PCVA: physician-certified verbal autopsy; *ρ*_C_: Lin’s concordance correlation coefficient

Using study-defined codes, the clinicopathological panel assigned 14/34 (41.2%) individuals a ‘TB in an HIV-positive individual’ CoD and 15 (44.1%) an ‘HIV/AIDS-related, excluding TB’ CoD (Table 2; S3 Table); PCVA assigned these CoD to 11 (32.4%) and 17 (50%) individuals, respectively. Agreement between PCVA and L3, at an individual level, was very poor (Κ 0.04; overall CCC 0.04; Table 3); agreement was better at a population level (*ρ*_C_ 0.91; CSMF accuracy 0.79).

### Performance of VA against L2 reference standard

Using ICD-10 codes, an HIV/AIDS reference CoD was assigned to 183/259 (70.7%) individuals, to 206 (79.5%) by PCVA, to 48.1% by InterVA-4, and to 75 (29%) by SmartVA-Analyze (Table 4). One (0.4%), 14.3%, and five (1.9%) individuals were assigned a ‘pulmonary TB’ CoD by PCVA, InterVA-4, and SmartVA-Analyze, respectively. All VA methods performed poorly when individual agreement was measured: K 0.06, 0.08, and 0.05; overall CCC 0.14, 0.06, and 0.15, for PCVA, InterVA-4, and SmartVA-Analyze, respectively (Table 3). PCVA performed best at population level (*ρ*_C_ 0.93; CSMF accuracy 0.78), InterVA-4 second-best (*ρ*_C_ 0.78; CSMF accuracy 0.61), and SmartVA-Analyze least well (*ρ*_C_ 0.52; CSMF accuracy 0.57). Using study-defined codes, physicians assigned 69/259 (26.6%) individuals a reference CoD of ‘TB in an HIV-positive individual’ and 103 (39.8%) individuals an ‘HIV/AIDS-related, excluding TB’ CoD (Table 4). PCVA agreement with L2 was poor at an individual level (K 0.03; overall CCC 0.08) but better at population level (*ρ*_C_ 0.70; CSMF accuracy 0.71).

**Table 4:** Numbers and resultant CSMFs generated for grouped ICD-10 and study-defined CoD categories, as assigned by L2 and L1 clinical panels, PCVA, InterVA-4, and SmartVA-Analyze (n = 259).

CoD category	Clinical panel		VA method		
	L2, n (%)	L1, n (%)	PCVA, n (%)	InterVA-4, % (CSMF)*	SmartVA-Analyze, n (%)
**Grouped ICD-10**					
HIV/AIDS	183 (70.7)	143 (55.2)	206 (79.5)	48.1	75 (29.0)
PTB	0	0	1 (0.4)	14.3	5 (1.9)
Other infectious	2 (0.8)	4 (1.5)	17 (6.6)	9.7	26 (10.0)
Malignancy	8 (3.1)	8 (3.1)	7 (2.7)	13.7	58 (22.4)
Other NCD	7 (2.7)	9 (3.5)	21 (8.1)	7.9	34 (13.1)
External/traumatic or pregnancy-related	3 (1.2)	3 (1.2)	7 (2.7)	1.2	1 (0.4)
Indeterminate	56 (21.7)	92 (35.5)	0	5.1	60 (23.2)
**Study-defined**					
TB in an HIV-positive individual	69 (26.6)	56 (21.6)	109 (42.1)	-	-
HIV-related cause other than TB	103 (39.8)	82 (31.7)	110 (42.5)	-	-
Other cause in an HIV-positive individual	18 (6.9)	16 (6.2)	20 (7.7)	-	-
TB in an HIV-negative individual	0	0	1 (0.4)	-	-
Other cause in an HIV-negative individual	0	0	19 (7.3)	-	-
Indeterminate cause	69 (26.6)	105 (40.5)	0	-	-

* InterVA-4 CSMFs generated from all assigned CoD and associated likelihoods

AIDS: acquired immune deficiency syndrome; CoD: cause of death; CSMF: cause-specific mortality fraction; HIV: human immunodeficiency virus; ICD: International Classification of Diseases; L1: level one (operational data only); L2: level two (operational and research data); NCD: non-communicable disease; PCVA: physician-certified verbal autopsy; PTB: pulmonary tuberculosis; TB: tuberculosis

### Cause-specific comparisons

Compared to L3 and L2, PCVA was more sensitive, but less specific, than both CCVA methods in assigning ICD-10 HIV/AIDS-related CoD (sensitivity against L3 75.0%, 50.0%, and 40.6%; sensitivity against L2 80.9%, 55.7%, and 31.7%; and specificity against L2 23.7%, 64.5% and 77.6% for PCVA, InterVA-4, and SmartVA-Analyze, respectively; Table 5). Compared to L2, PCVA was only 44.9% sensitive and 58.9% specific for a study-defined CoD of ‘TB in an HIV-positive individual’, with CCC measured at 0.42; sensitivity and CCC were lower when compared to L3, at 35.7% and 0.23, respectively.

**Table 5:** Sensitivity, specificity, and chance-corrected concordance of PCVA, InterVA-4, and SmartVA-Analyze in the detection of specific CoD compared to L3 (n = 34) and L2 (n = 259) clinical reference standards.

CoD category and VA method	Compared to L3 (n = 34)			Compared to L2 (n = 259)		
	Sensitivity, % (95% CI)	Specificity, % (95% CI)	CCC	Sensitivity, % (95% CI)	Specificity, % (95% CI)	CCC
**HIV/AIDS (ICD-defined)**						
PCVA	75.0 (56.6–88.5)	50.0 (1.3–98.7)	0.71	80.9 (74.4–86.3)	23.7 (14.7–34.8)	0.78
InterVA-4*	50.0 (31.9–68.1)	100.0 (15.8–100.0)	0.42	55.7 (48.2–63.1)	64.5 (52.7–75.1)	0.48
SmartVA-Analyze	40.6 (23.7–59.4)	50.0 (1.3–98.7)	0.31	31.7 (25.0–39.0)	77.6 (66.6–86.4)	0.20
**TB in an HIV-positive individual (study-defined)**						
PCVA	35.7 (12.8–64.9)	70.0 (45.7–88.1)	0.23	44.9 (32.9–57.4)	58.9 (51.6–66.0)	0.42
**HIV-related cause other than TB (study-defined)**						
PCVA	53.3 (26.6–78.7)	52.6 (28.9–75.6)	0.44	26.2 (17.2–36.9)	77.1 (70.2–83.1)	0.36

* Measures of InterVA-4 sensitivity and specificity calculated using individuals assigned HIV/AIDS as ‘most likely’ CoD (n = 129 overall; n = 16 with L3 CoD)

AIDS: acquired immune deficiency syndrome; CCC: chance-corrected concordance; CI: confidence interval; CoD: cause of death; HIV: human immunodeficiency virus; ICD: International Classification of Diseases; L2: level two cause of death (operational and research data); L3: level three cause of death (operational, research, and autopsy data); PCVA: physician-certified verbal autopsy; TB: tuberculosis; VA: verbal autopsy

## Discussion

This study, conducted among HIV-positive adults in a setting of high TB prevalence, compared CoD assigned by VA to a robust gold standard, including MIA, and found the proportion of deaths attributable to TB was underestimated by methods that did not include data from pathological autopsy. Overall HIV-associated mortality was also underestimated by all VA methods when compared to the L3 (autopsy) reference standard, with poor agreement at an individual level; all methods performed better at a population level.

### HIV-associated TB

A recent systematic review of autopsy studies in HIV-positive individuals found that 46% of TB diagnosed at autopsy had not been diagnosed ante-mortem and that almost 90% of HIV-positive individuals with evidence of TB at autopsy had disseminated disease [4]. In our study, every individual with MIA data assigned a reference-standard CoD of HIV-associated TB had evidence of extrapulmonary and/or disseminated disease at post-mortem (S3 Table); 6/16 (38%) individuals with evidence of active TB at autopsy had not been started on TB treatment between enrolment and death. The under-diagnosis of TB in the absence of autopsy data suggests that the long-standing emphasis on respiratory symptoms and sputum-based investigation make it less likely for physicians to consider a TB diagnosis in patients who do not report a cough. This has important implications for the development of TB diagnostics, the design of guidelines, and in the training of clinicians operating in areas of high HIV prevalence. It may also mean that current CCVA algorithms used to generate VA CoD need recalibration to account for those with advanced HIV and extrapulmonary or disseminated TB, who may have few or no respiratory symptoms.

### ICD-10 coding of HIV-deaths

ICD-10 coding does not differentiate HIV-associated TB death from other HIV-associated causes, therefore deaths due to HIV-associated TB are effectively ‘hidden’ within HIV-related deaths a whole. TB is the leading cause of death in HIV-positive individuals and being unable to directly measure mortality attributable to it is an enormous disadvantage. As the global ART rollout continues and the HIV epidemic evolves, it is no longer sufficient to talk simply about deaths due to HIV; a more nuanced approach to disease and mortality measurement is needed [10,37] and central to this approach must be modifications to the ICD system to allow for differentiation of HIV-associated TB deaths from other HIV-related deaths. The current draft of ICD-11, due for release in 2018 [38,39], allows for the separation of HIV disease by clinical stage and for the inclusion of certain co-morbidities, such as TB and malaria. A separate three-character code denoting HIV-associated TB would be a welcome addition to these developments.

### Comparison to previous studies

To our knowledge, only one other study, conducted in Kenya, has attempted to compare VA CoD to CoD derived from pathological autopsy. In this study both PCVA and InterVA-4, when compared to MIA, overestimated mortality due to TB in an area of high HIV prevalence [11]. However, in addition to HIV-positive adults, this study included children and HIV-negative individuals and selected only individuals who reported respiratory symptoms. This may have led to the exclusion of those with extrapulmonary TB and no respiratory symptoms and may account for the relatively low prevalence of TB seen at pathological autopsy. Our findings suggest the opposite, that VA underestimates deaths due to TB, but we would nevertheless agree with the authors of the Kenyan study that, at present, VA is not a suitable tool for assigning individual CoD in areas of high HIV prevalence and would be cautious about its use in registering individual deaths [40–42].

A number of studies have attempted to use VA to estimate CSMFs in areas of high HIV prevalence using a variety of gold standards. Most studies grouped HIV-associated TB deaths with other HIV/AIDS deaths, as per ICD-10 [10,22,43–51], or did not clearly differentiate between ‘TB’ and ‘HIV/AIDS’ categories [52–56]. At least some of this inconsistency is due to the issues with ICD coding discussed above. For example, one of the largest studies, an analysis of 54,000 deaths from the International Network for the Demographic Evaluation of Populations and Their Health (INDEPTH) HDSS sites in Africa and Asia, compared InterVA-4 to a PCVA gold standard; *ρ*_C_ was 0.831 overall and increased to 0.974 when HIV/AIDS and pulmonary TB CoD were combined for deaths from sub-Saharan Africa [57]. This high figure is misleading, however, as the two categories are intended to be mutually exclusive [58]; classifying HIV-associated TB deaths as ‘pulmonary TB’ will lead to the overall underestimation of HIV-associated deaths if current ICD rules are correctly applied. In our study, 35/41 (85.4%) individuals assigned a ‘PTB’ CoD by InterVA-4 were reported HIV-positive during the VA interview, but 32/35 (91%) did not have HIV/AIDS mentioned as a second or third CoD. Another important issue is that of extrapulmonary and disseminated disease: the WHO truncated CoD list classifies extrapulmonary and disseminated TB (ICD-10 codes A17–A19) under ‘Other or unspecified infectious diseases’ [59]; even if PTB and HIV/AIDS categories were combined for analysis purposes, the exclusion of these forms of TB would still result in the underestimation of TB-related deaths.

Only one previous study, conducted in 1998 across sites in Tanzania, Ethiopia, and Ghana, attempted to use VA to differentiate HIV-associated TB from other HIV-associated CoD, comparing CoD from PCVA and an early CCVA algorithm to CoD derived from hospital diagnoses [24]. Similar to our findings, both VA analysis methods showed low sensitivity and high specificity for ‘TB + AIDS’ diagnoses (respectively, 8% and 99% for PCVA and 35% and 95% for the CCVA algorithm), with PCVA detecting only 11/35 (31%) cases of ‘TB + AIDS’. More recently, in the construction of the PHMRC gold standard dataset, CoD were initially classified as ‘AIDS’; ‘AIDS with TB’; or ‘Pulmonary TB’, with criteria explicitly stated for each [32]. The categories were consistent with ICD-10: inclusion in the ‘Pulmonary TB’ category required the individual to have tested HIV-negative. However, to be included in the ‘AIDS with TB’ category, an individual was required to have both a positive HIV test and a positive culture for *M*. *tuberculosis*, which likely led to the exclusion of individuals with disseminated TB and limited or no respiratory symptoms. To date, all comparisons of VA to the PHMRC dataset, including those conducted by the PHMRC team, have combined the ‘AIDS’ and ‘AIDS with TB’ categories, and have therefore not attempted to assess VA’s ability to detect HIV-associated TB [19,20,30,60–64]. The PHMRC gold standard dataset nevertheless remains a valuable resource; we would suggest that any future validation exercises use the differentiated, ‘AIDS with TB’ and ‘AIDS’ categories, rather than the combined ‘AIDS’ category, for comparison to VA.

### Moving forward

In the absence of robust, validated CRVS data, there are few alternatives to VA that are both feasible and cost-effective in generating estimates of cause-specific mortality in countries with high HIV and TB prevalence [65,66]. Although, in this study, VA methods performed poorly in assigning individual CoD, it should be noted that VA is primarily intended to generate population-level estimates [18], and that performance in this regard was better. However, when using study-defined codes, which were designed to allow for the differentiation of HIV-associated TB from other HIV-associated causes, the population-level accuracy of PCVA was still sub-optimal (*ρ*_C_ 0.70 and CSMF accuracy 0.71 compared to L2 standard [n = 259]; Table 3), confirming the difficulty of making this distinction.

The challenges of diagnosing HIV-associated TB disease are well documented [67,68] and, as found in the systematic review of autopsy studies [4], in the absence of new diagnostics it is likely that clinicians will continue to underdiagnose TB, which will have important implications for measuring progress towards the WHO targets described above [16]. Improvements are needed to TB surveillance methods, which, at present, consist mostly of enumerating individuals already diagnosed and started on treatment [69–71]. MIA is a useful technique for estimating the prevalence of infectious diseases [72,73], is acceptable to a high proportion of families [26,74], and could be used periodically for surveillance at sentinel sites [75], allowing for more accurate evaluation of the impact of disease-focused interventions.

Population-level estimates of cause-specific mortality are extremely valuable and improving the accuracy of VA-generated estimates would be of benefit, regardless of whether or not VA is used to assign individual CoD. The continued development and sharing of gold standard datasets that include pathological autopsy data, better reflecting the high proportions of HIV-associated mortality seen in high-burden countries and including both hospital and community deaths in different populations, would allow for greater standardisation in future validation studies. The parallel development of a structured, standardised process for CoD assignment, similar to that described in the Coding Causes of Death in HIV (CoDe) project [76], but assigning CoD matched to ICD codes [77], would increase the value of this exercise.

### Limitations and strengths

This study had limitations: the median time from death to VA was slightly longer than the ideal three months that some recommend, but was well within the maximum 12 months recommended by WHO and was therefore considered unlikely to have had a substantial effect on VA-generated estimates [78,79]; physicians who reviewed clinical and VA data were aware that most decedents were likely HIV-positive and had been enrolled into TB-focused studies, which may have led to greater assignment of HIV- and TB-related CoD; missing operational and research data may have affected consistency of the reference standard; pathological autopsy data were available for a small number of decedents; and, although the reference CoD assigned represent our best estimates using the data available, the true CoD may still differ. Questions on ART and TB treatment, added to the VA instrument by the study team, may have led to changes in how events were reported in the free narrative section; the answers to the questions themselves, however, were not provided to reviewing physicians or to either software. InterVA-4 and SmartVA-Analyze are designed for use with the WHO 2014 and PHMRC VA instruments, respectively, therefore using the WHO 2012 instrument may have resulted in some missing variables; healthcare and narrative data were not provided to SmartVA-Analyze, which may have affected its assignment of CoD. Individuals included in this analysis are likely representative of those with advanced HIV disease in resource-scarce settings, but may not necessarily represent the patterns of mortality seen in the wider community. This may have affected measures of agreement that are dependent on the composition of the gold standard CSMF and may, in turn, limit the generalisability of our findings. This study’s strengths include: having recent, reliable information regarding CD4 count, ART status, and investigation and treatment of TB; using the same physicians to assign PCVA CoD for all decedents; using robust methods to assign reference CoD; comparing VA-assigned CoD to a reference standard that included MIA findings; comparing between CoD using a range of metrics, allowing for evaluation of different potential applications of VA; and classifying HIV-associated TB separately from other HIV-associated CoD, something made difficult by ICD-10 and generally neglected by previous VA studies.

## Conclusions

Current VA methods underestimate mortality due to HIV-associated TB. At present, VA does not assign individual CoD in areas of high HIV prevalence with sufficient accuracy and, in part due to the limitations of ICD-10, does not distinguish between deaths due to HIV-associated TB and advanced HIV disease. More accurate methods are needed that allow for direct estimation of deaths due to HIV-associated TB; unless TB mortality is more accurately measured, it will be extremely difficult to track progress towards the goals set by the post-2015 global strategy.

## Supporting information

S1 Fig Numbers of individuals enrolled to each of the parent studies, number of deaths, number of VAs conducted, data sources for clinical cause of death assignment, data availability by parent study, and which data contributed to different CoD levels. (TIF)

S1 Table Grouped ICD-10 CoD categories and corresponding WHO 2014 CoD codes. (DOCX)

S2 Table Criteria used by clinical panels to assign study-defined CoD and associated certainty. (DOCX)

S3 Table Causes of Death (CoD) for decedents with 'Autopsy' data: ICD-10 immediate and underlying CoD, as assigned by reviewers; grouped ICD-10 category; and study-specific categories as assigned by clinicopathological panel (L3), physician-certified VA, InterVA-4, and SmartVA-Analyze (n = 34). (DOCX)
