# Supplementary figures and images for: Measuring mortality due to HIV-associated tuberculosis among adults in South Africa: Comparing verbal autopsy, minimally-invasive autopsy, and research data
Source: PLoS One. 2017 Mar 23;12(3):e0174097. doi: 10.1371/journal.pone.0174097 (PMC5363862; doi:10.1371/journal.pone.0174097)

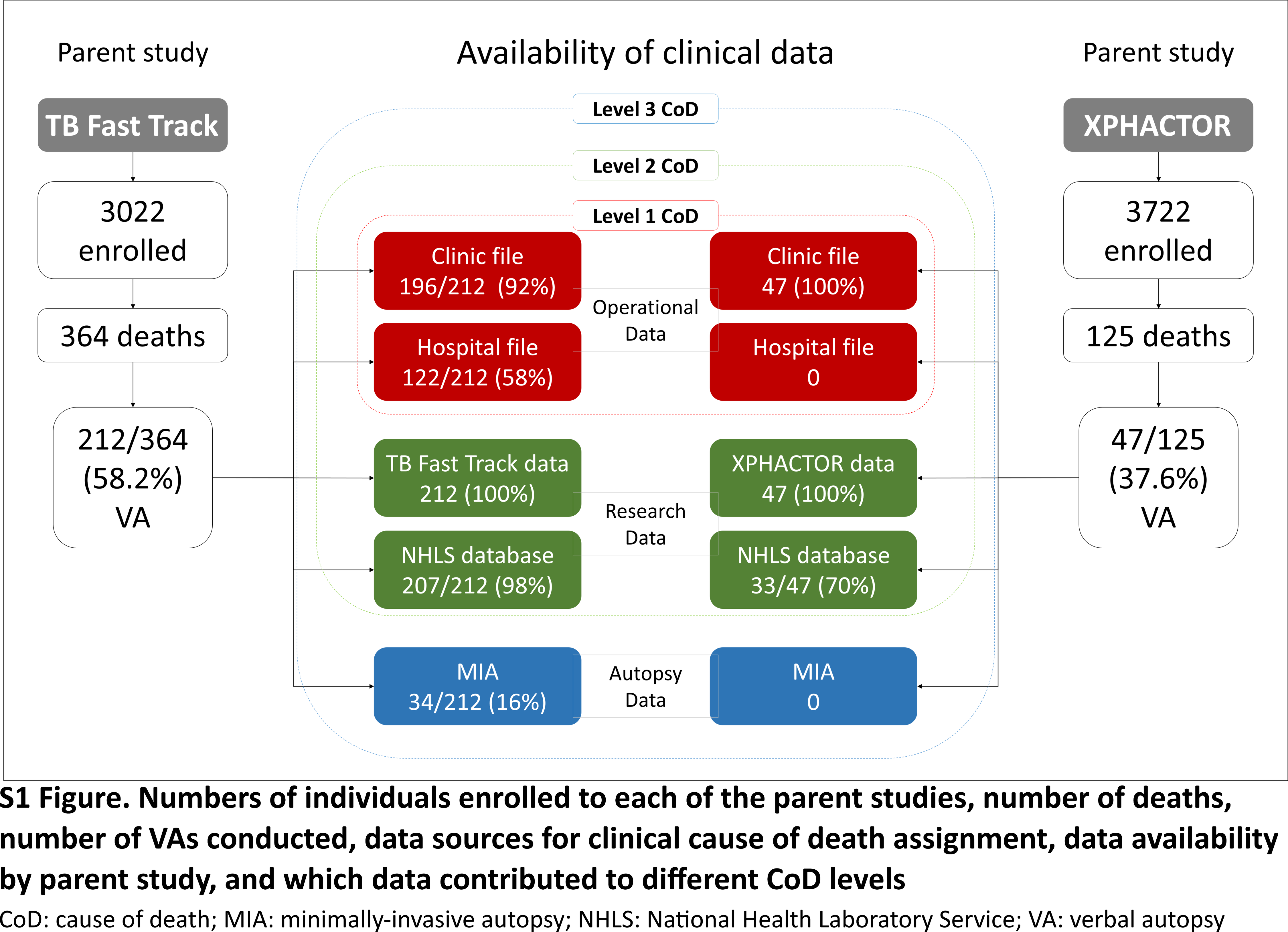

Supplement: S1 Fig — (TIF) [file pone.0174097.s001.tif]
