# Supplementary material for: Measuring mortality due to HIV-associated tuberculosis among adults in South Africa: Comparing verbal autopsy, minimally-invasive autopsy, and research data
Source: PLoS One. 2017 Mar 23;12(3):e0174097. doi: 10.1371/journal.pone.0174097 (PMC5363862; doi:10.1371/journal.pone.0174097)
Supplement: S1 Table — (DOCX) [file pone.0174097.s002.docx]

Supporting table 1: Grouped ICD-10 CoD categories and corresponding WHO 2014 CoD codes

| **Grouped ICD-10 category*** | **Corresponding WHO 2014 CoD codes** [56] |
| --- | --- |
| HIV/AIDS-related | 01.03 HIV/AIDS-related death |
| Pulmonary TB | 01.09 Pulmonary tuberculosis |
| Other infections | 01.01 Sepsis (non-obstetric) |
|  | 01.02 Acute resp. infection, incl. pneumonia |
|  | 01.04 Diarrhoeal illness |
|  | 01.05 Malaria |
|  | 01.06 Measles |
|  | 01.07 Meningitis and encephalitis |
|  | 01.08, 10.05 Tetanus |
|  | 01.10 Pertussis |
|  | 01.11 Haemorrhagic fever |
|  | 01.99 Other and unspecified infectious diseases |
| Non-HIV malignancy | 02.01 Oral neoplasms |
|  | 02.02 Digestive neoplasms |
|  | 02.03 Respiratory neoplasms |
|  | 02.04 Breast neoplasms |
|  | 02.05, 02.06 Reproductive neoplasms M, F |
|  | 02.99 Other and unspecified neoplasms |
| Other non-communicable diseases | 03.01 Severe anaemia |
|  | 03.02 Severe malnutrition |
|  | 03.03 Diabetes mellitus |
|  | 04.01 Acute cardiac disease |
|  | 04.02 Stroke |
|  | 04.03 Sickle cell with crisis |
|  | 04.99 Other and unspecified cardiac disease |
|  | 05.01 Chronic obstructive pulmonary disease |
|  | 05.02 Asthma |
|  | 06.01 Acute abdomen |
|  | 06.02 Liver cirrhosis |
|  | 07.01 Renal failure |
|  | 08.01 Epilepsy |
|  | 98 Other and unspecified NCD |
| External / Traumatic / Pregnancy | 09.01 Ectopic pregnancy |
|  | 09.02 Abortion-related death |
|  | 09.03 Pregnancy-induced hypertension |
|  | 09.04 Obstetric haemorrhage |
|  | 09.05 Obstructed labour |
|  | 09.06 Pregnancy-related sepsis |
|  | 09.07 Anaemia of pregnancy |
|  | 09.08 Ruptured uterus |
|  | 09.99 Other and unspecified maternal CoD |
|  | 12.01 Road traffic accident |
|  | 12.02 Other transport accident |
|  | 12.03 Accidental fall |
|  | 12.04 Accidental drowning and submersion |
|  | 12.05 Accidental exposure to smoke, fire, & flame |
|  | 12.06 Contact with venomous plant/animal |
|  | 12.07 Accidental poisoning and noxious substances |
|  | 12.08 Intentional self-harm |
|  | 12.09 Assault |
|  | 12.10 Exposure to force of nature |
|  | 12.99 Other and unspecified external CoD |
| Indeterminate | Cause of death unknown |
| *Categories used for analysis purposes  AIDS: Acquired immune deficiency syndrome; CoD: cause of death; F: Female; HIV: Human immunodeficiency virus; ICD: International Classification of Diseases; M: Male; NCD: Non-communicable disease; TB: tuberculosis; WHO: World Health Organization | |
