# Supplementary material for: Measuring mortality due to HIV-associated tuberculosis among adults in South Africa: Comparing verbal autopsy, minimally-invasive autopsy, and research data
Source: PLoS One. 2017 Mar 23;12(3):e0174097. doi: 10.1371/journal.pone.0174097 (PMC5363862; doi:10.1371/journal.pone.0174097)
Supplement: S2 Table — (DOCX) [file pone.0174097.s003.docx]

Supporting table 2: Criteria used by clinical panels to assign study-defined CoD and associated certainty

| **Study-defined category** | **Certainty** | | |
| --- | --- | --- | --- |
|  | **‘Definite’** | **‘Probable’** | **‘Possible’** |
| Death due to TB in an HIV positive individual | Documented evidence of positive HIV point-of-care test or ELISA **OR** patient on ART | Documented evidence of positive HIV point-of-care test or ELISA **OR** patient on ART | History suggestive of HIV and active TB, but no clear evidence available |
|  | **AND** | **AND** |  |
|  | History and clinicopathological features consistent with active TB disease | History suggestive of active TB disease |  |
|  | **AND** | **AND** |  |
|  | Microbiological evidence of TB  (culture positive or Xpert® MTB/RIF positive) | Positive sputum smear, urine LAM or chest x-ray |  |
| Death due to other HIV/AIDS-associated cause (excluding TB) | Documented evidence of positive HIV point-of-care test or ELISA **OR** patient on ART | Documented evidence of positive HIV point-of-care test or ELISA **OR** patient on ART | History suggestive of an AIDS-defining or HIV-associated condition (other than TB) in a person thought to be HIV-positive or a condition thought to be due to ART |
|  | **AND** | **AND** |  |
|  | History and clinicopathological findings consistent with an AIDS-defining or HIV-associated condition (other than TB), or a condition due to ART (only applicable if ART use documented) | History consistent with an AIDS-defining or HIV-associated condition (other than TB), or a condition likely due to ART (only applicable if ART use suggested) |  |
|  | **AND** | **AND** |  |
|  | Microbiological/histological/cytological evidence for one of the above | Documentation of one of the above conditions by a senior clinician |  |
| Death due to cause unrelated to HIV in an HIV-positive adult | Documented evidence of positive HIV point-of-care test or ELISA **OR** patient on ART | Documented evidence of positive HIV point-of-care test or ELISA **OR** patient on ART | Documented evidence of positive HIV point-of-care test or ELISA **OR** patient on ART |
|  | **AND** | **AND** | **AND** |
|  | History and clinicopathological findings consistent with a condition unrelated to HIV | History consistent with a condition unlikely to be related to HIV | History suggestive of a condition unlikely to be related to HIV |
| Death due to TB in an HIV-negative adult | Documented evidence of negative HIV point-of-care test or ELISA in the 90 days prior to death | Documented HIV-negative point-of-care test or ELISA over 90 days but less than 180 days prior to death) | Patient self-reports HIV negative |
|  | **AND** | **AND** | **AND** |
|  | History consistent with active TB disease of a severity sufficient to cause death | History suggestive of active TB disease of a severity sufficient to cause death | No other evidence of HIV disease or an HIV test |
|  | **AND** | **AND** | **AND** |
|  | Microbiological evidence of TB (culture positive, Xpert® MTB/RIF positive) | Positive sputum smear or urine LAM or chest x-ray | History suggestive of active TB disease (of a severity sufficient to cause death) |
| Death due to cause other than TB in an HIV-negative adult | Documented evidence of negative HIV point-of-care test or ELISA in the 90 days prior to death | Documented HIV-negative point-of-care test or ELISA over 90 days but less than 180 days prior to death) | Patient self-reports HIV negative |
|  |  |  | **AND** |
|  | **AND** | **AND** | No other evidence of HIV disease or HIV test |
|  | No evidence (history or investigations) of TB | No evidence (history or investigations) of TB  **OR**  Possible evidence of TB disease (e.g.: suggestive symptoms) but clinical history not consistent | **AND** |
|  |  |  | No evidence (history or investigations) of TB  **OR**  Possible evidence of TB infection/previous exposure but clinical history not consistent |
| Death due to indeterminate cause | Cause of death unclear | | |
| AIDS: Acquired immune deficiency syndrome; ART: antiretroviral therapy; CoD: cause of death; ELISA: Enzyme-linked immunosorbent assay; HIV: human immunodeficiency virus; LAM: lipoarabinomannan; TB: tuberculosis | | | |
