# Supplementary material for: Measuring mortality due to HIV-associated tuberculosis among adults in South Africa: Comparing verbal autopsy, minimally-invasive autopsy, and research data
Source: PLoS One. 2017 Mar 23;12(3):e0174097. doi: 10.1371/journal.pone.0174097 (PMC5363862; doi:10.1371/journal.pone.0174097)
Supplement: S3 Table — (DOCX) [file pone.0174097.s004.docx]

Supporting table 3. CoD for decedents with 'Autopsy' data: ICD-10 immediate and underlying CoD, as assigned by reviewers; grouped ICD-10 category; and study-specific categories as assigned by clinicopathological panel (L3), PCVA, InterVA-4, and SmartVA-Analyze (n=34)

| **ID†** | **Level three CoD** | | | | **Physician-certified VA CoD** | | | | **InterVA-4 CoD** | **SmartVA-Analyze CoD** |
| --- | --- | --- | --- | --- | --- | --- | --- | --- | --- | --- |
|  | **‘Immediate’** | **‘Underlying’** | **Grouped ICD-10** | **Study-defined** | **‘Immediate’** | **‘Underlying’** | **Grouped ICD-10** | **Study-defined** | **Grouped ICD-10** | |
| **1** | HIV disease |  | HIV/AIDS-related | Indeterminate | PTB | HIV disease | HIV/AIDS-related | TB in HIV-positive | HIV/AIDS-related | HIV/AIDS-related |
| **2** | Disseminated TB | HIV disease | HIV/AIDS-related | TB in HIV-positive | PTB | HIV disease | HIV/AIDS-related | TB in HIV-positive | Pulmonary TB | HIV/AIDS-related |
| **3** | Disseminated TB | HIV disease | HIV/AIDS-related | TB in HIV-positive | PTB | HIV disease | HIV/AIDS-related | TB in HIV-positive | HIV/AIDS-related | Other NCD |
| **4** | Cryptococcal disease | HIV disease | HIV/AIDS-related | HIV/AIDS, excl. TB | PTB | HIV disease | HIV/AIDS-related | TB in HIV-positive | HIV/AIDS-related | HIV/AIDS-related |
| **5** | Gastroenteritis | HIV disease | Other infectious | HIV/AIDS, excl. TB | Stroke | HIV disease | HIV/AIDS-related | HIV/AIDS, excl. TB | Other NCD | Other infectious |
| **6** | Disseminated TB | HIV disease | HIV/AIDS-related | TB in HIV-positive | Bacterial meningitis | HIV disease | HIV/AIDS-related | HIV/AIDS, excl. TB | Non-HIV malignancy | HIV/AIDS-related |
| **7** | Disseminated TB | HIV disease | HIV/AIDS-related | TB in HIV-positive | Bacterial infection | HIV disease | HIV/AIDS-related | HIV/AIDS, excl. TB | HIV/AIDS-related | Other NCD |
| **8** | Disseminated TB | HIV disease | HIV/AIDS-related | TB in HIV-positive | Disseminated TB | HIV disease | HIV/AIDS-related | TB in HIV-positive | HIV/AIDS-related | HIV/AIDS-related |
| **9** | Bacterial pneumonia | HIV disease | HIV/AIDS-related | TB in HIV-positive | PTB | HIV disease | HIV/AIDS-related | TB in HIV-positive | Pulmonary TB | HIV/AIDS-related |
| **10** | Salmonellosis | HIV disease | HIV/AIDS-related | HIV/AIDS, excl. TB | HIV disease |  | HIV/AIDS-related | HIV/AIDS, excl. TB | Other infectious | Non-HIV malignancy |
| **11** | PCP | HIV disease | HIV/AIDS-related | HIV/AIDS, excl. TB | Gastroenteritis | HIV disease | Other infectious | HIV/AIDS, excl. TB | HIV/AIDS-related | HIV/AIDS-related |
| **12** | CNS disorder | HIV disease | HIV/AIDS-related | Indeterminate | Stroke | HIV disease | HIV/AIDS-related | Non-HIV in HIV-positive | Other NCD | Indeterminate |
| **13** | Bacterial pneumonia | HIV disease | HIV/AIDS-related | HIV/AIDS, excl. TB | PTB | HIV disease | HIV/AIDS-related | TB in HIV-positive | Pulmonary TB | HIV/AIDS-related |
| **14** | Disseminated TB | HIV disease | HIV/AIDS-related | TB in HIV-positive | Gastroenteritis | HIV disease | Other infectious | HIV/AIDS, excl. TB | Other infectious | Other infectious |
| **15** | LRTI | HIV disease | HIV/AIDS-related | Indeterminate | HIV disease |  | HIV/AIDS-related | HIV/AIDS, excl. TB | HIV/AIDS-related | Other NCD |
| **16** | Disseminated TB | HIV disease | HIV/AIDS-related | TB in HIV-positive | Peptic ulcer | EPTB | Other NCD | TB in HIV-positive | Non-HIV malignancy | Non-HIV malignancy |
| **17** | Indeterminate | HIV disease | HIV/AIDS-related | Indeterminate | PTB | HIV disease | HIV/AIDS-related | TB in HIV-positive | Other NCD | HIV/AIDS-related |
| **18** | Disseminated TB | HIV disease | HIV/AIDS-related | TB in HIV-positive | HIV disease |  | HIV/AIDS-related | HIV/AIDS, excl. TB | HIV/AIDS-related | HIV/AIDS-related |
| **19** | Cryptococcal disease | HIV disease | HIV/AIDS-related | HIV/AIDS, excl. TB | PE | HIV disease | HIV/AIDS-related | Non-HIV in HIV-positive | Non-HIV malignancy | Indeterminate |
| **20** | Disseminated TB | HIV disease | HIV/AIDS-related | TB in HIV-positive | Liver disease | HIV disease | HIV/AIDS-related | HIV/AIDS, excl. TB | HIV/AIDS-related | Indeterminate |
| **21** | Bacterial pneumonia | HIV disease | HIV/AIDS-related | HIV/AIDS, excl. TB | Pneumonia | Type two diabetes | Other infectious | Non-TB in HIV-negative | Other infectious | Other NCD |
| **22** | Bacterial pneumonia | HIV disease | HIV/AIDS-related | HIV/AIDS, excl. TB | Gas gangrene | HIV disease | HIV/AIDS-related | Non-HIV in HIV-positive | Non-HIV malignancy | Other NCD |
| **23** | NTM disease | HIV disease | HIV/AIDS-related | HIV/AIDS, excl. TB | Chronic renal failure | HIV disease | HIV/AIDS-related | HIV/AIDS, excl. TB | HIV/AIDS-related | Other infectious |
| **24** | NTM disease | HIV disease | HIV/AIDS-related | HIV/AIDS, excl. TB | PTB | HIV disease | HIV/AIDS-related | TB in HIV-positive | Pulmonary TB | Indeterminate |
| **25** | Transport accident |  | External/trauma | Non-HIV in HIV-positive | Injuries to spine and trunk | Transport accident | External/trauma | Non-TB in HIV- negative | Pulmonary TB | HIV/AIDS-related |
| **26** | EPTB | HIV disease | HIV/AIDS-related | TB in HIV-positive | GI obstruction |  | Other NCD | Non-TB in HIV-negative | Other NCD | Indeterminate |
| **27** | NTM disease | HIV disease | HIV/AIDS-related | HIV/AIDS, excl. TB | HIV disease |  | HIV/AIDS-related | HIV/AIDS, excl. TB | HIV/AIDS-related | Other infectious |
| **28** | Disseminated TB | HIV disease | HIV/AIDS-related | TB in HIV-positive | HIV disease |  | HIV/AIDS-related | HIV/AIDS, excl. TB | HIV/AIDS-related | HIV/AIDS-related |
| **29** | Nocardiosis | HIV disease | HIV/AIDS-related | HIV/AIDS, excl. TB | HIV disease |  | HIV/AIDS-related | HIV/AIDS, excl. TB | HIV/AIDS-related | HIV/AIDS-related |
| **30** | Pneumonia | HIV disease | HIV/AIDS-related | HIV/AIDS, excl. TB | HIV disease |  | HIV/AIDS-related | HIV/AIDS, excl. TB | HIV/AIDS-related | 4. Indeterminate |
| **31** | Disseminated TB | HIV disease | HIV/AIDS-related | TB in HIV-positive | Gastroenteritis | HIV disease | Other infectious | HIV/AIDS, excl. TB | HIV/AIDS-related | Other infectious |
| **32** | Disseminated TB | HIV disease | HIV/AIDS-related | TB in HIV-positive | Bacterial pneumonia | HIV disease | HIV/AIDS-related | HIV/AIDS, excl. TB | Pulmonary TB | 4. Indeterminate |
| **33** | CMV | HIV disease | HIV/AIDS-related | HIV/AIDS, excl. TB | Hepatic failure | PTB | Other NCD | TB in HIV-positive | HIV/AIDS-related | HIV/AIDS-related |
| **34** | PCP | HIV disease | HIV/AIDS-related | HIV/AIDS, excl. TB | Hepatic failure | PTB | Other NCD | HIV/AIDS, excl. TB | Pulmonary TB | PTB |
| †ID denotes chronological order in which participants died  AIDS: Acquired immune deficiency syndrome; CMV: cytomegalovirus; CNS: central nervous system; CoD: cause of death; excl.: excluding; EPTB: Extrapulmonary tuberculosis; GI: gastrointestinal; HIV: Human immunodeficiency virus; ICD: International Classification of Diseases; L3: level three reference cause of death (‘operational’, ‘research’, and ‘autopsy’ data); LRTI: lower respiratory tract infection; NCD: non-communicable disease; NTM: Non-tuberculous mycobacteria; PCP: *Pneumocystis* pneumonia; PCVA: physician-certified verbal autopsy; PE: pulmonary embolism; PTB: pulmonary tuberculosis; TB: tuberculosis; VA: verbal autopsy | | | | | | | | | | |
